# Study of the Immunohistochemical Expression of p63 in Benign Lesions and Carcinoma of the Breast at a Tertiary Hospital in South India

**DOI:** 10.7759/cureus.48557

**Published:** 2023-11-09

**Authors:** Shreya Dinesh Prabhu, Hassan Sona Rai, Rakshatha Nayak, Ramadas Naik, Shikha Jayasheelan

**Affiliations:** 1 Pathology, Rajarajeswari Medical College and Hospital, Bengaluru, IND; 2 Pathology, BGS Global Institute of Medical Sciences, Bengaluru, IND; 3 Pathology, Kasturba Medical College, Mangalore, Manipal Academy of Higher Education, Manipal, IND; 4 Pathology, Yenepoya Medical College, Mangalore, IND

**Keywords:** myoepithelial cells, immunohistochemistry, invasive breast carcinoma, ductal carcinoma in situ, p63

## Abstract

Background: Invasive breast carcinoma is among the most common female cancers worldwide, causing high morbidity and mortality. Considerable disagreement in the interpretation of diagnostically challenging breast lesions based on histology alone has been documented. One of the essential histopathological findings that help distinguish benign from malignant lesions is the presence of the myoepithelial cell layer. Myoepithelial markers such as tumor protein 63 (p63) help distinguish invasive carcinoma from benign proliferations. p63 antibody is superior to other myoepithelial markers as it selectively stains the nuclei and is negative in stromal cells.

Objective: To study the expression of p63 in various histological subtypes and grades of breast carcinomas.

Methods: After routine hematoxylin and eosin stain, 65 cases of breast lesions were subjected to immunohistochemistry for p63 antigen using Novacastra ready-to-use monoclonal antibody p6. All cases were analyzed for p63 expression, and its staining arrangement was interpreted.

Results: In all benign lesions, immunoreactivity was noted in the myoepithelial cells, forming a continuous layer surrounding the luminal epithelial cells. The benign papillary lesions showed p63 staining in the fibrovascular core of the papillary fronds and at the periphery. A few single myoepithelial cells stained by p63 were also seen scattered discontinuously in ductal carcinoma in situ (DCIS). All invasive carcinomas and encapsulated papillary carcinomas were completely devoid of peripheral p63 staining of myoepithelial cells.

Conclusion: p63 is a specific nuclear marker of myoepithelial cells in the breast and can, therefore, aid in distinguishing invasive ductal carcinoma from DCIS or rare questionable hyperplastic lesions. They also play a significant role in distinguishing various papillary lesions of the breast and, hence, can be incorporated into routine reporting for definitive diagnosis and accurate treatment.

## Introduction

Protein 63 (p63), a member of the p53 family, is found on chromosome 3q27 [1]. The variable expression of p63 in human breast carcinomas suggests its possible role in tumorigenesis, metastasis, and prognosis [2]. The diagnoses of benign, papillary, and malignant proliferations of the breast can be achieved using hematoxylin and eosin microscopic sections alone in most cases. However, making this morphological distinction can be challenging in core needle biopsy, as well as in diagnosing certain conditions, such as borderline and atypical lesions, especially when there is inter-observer variability. Immunohistochemical (IHC) stains can, therefore, be helpful in diagnostics and in determining the prognosis of these lesions [3]. Three cell types expressing different protein subsets are seen in normal breast glands and ducts: luminal, basal, and myoepithelial. Different cytokeratins are described in the luminal and basal cell types, whereas myoepithelial cells express basal cell-type cytokeratins, smooth muscle actin, calponin, and p63. An intact myoepithelial layer is often seen in benign and in situ lesions, whereas loss of this layer is considered a diagnostic feature of invasive cancer [4-7]. p63 is a specific nuclear marker for myoepithelial cells of the breast with no known cross-reactivity and, therefore, may aid in differentiating benign lesions from malignant lesions [8]. Moreover, its variable expressions in different grades and histological subtypes of breast cancers have created a gap in the literature and, hence, require further elucidation of its expression in breast lesions [2,3].

## Materials and methods

This prospective study was conducted in the Department of Pathology at a tertiary care hospital in Southern India over a period of three years, from October 2013 to June 2015, after ethical clearance from the Yenepoya Medical College Institutional Ethical Committee (YUEC 113/15/11/2013). Clinical details were procured from the patients' histopathology requisition forms and the laboratory software. Biopsies, excision, tru-cut needle biopsy, and mastectomy specimens of both benign and malignant breast lesions were included in the study. Congenital breast diseases, inflammatory breast lesions, soft tissue lesions, metastatic deposits to the breast, and cases with prior treatment were excluded from the study. The specimens were fixed in 10% formalin, embedded in paraffin blocks, sectioned at 3-5μ, and stained with hematoxylin and eosin. They were studied under a light microscope and classified into benign lesions and non-invasive and invasive carcinomas. All cases were subjected to immunohistochemistry for p63 antigen using Novacastra ready-to-use monoclonal antibody, and the slides were studied for the presence of myoepithelial cells. Based on the pattern of staining and intensity of p63, the cases were graded as continuously positive with strong intensity, discontinuously positive with weak intensity, and negative. The sample size was calculated to be 65 using nMaster 2.0 software (Christian Medical College, Vellore, India). The chi-square test was used for quantitative analysis of data. Mean±SD and percentage were used to determine data for statistical analysis by using Statistical Product and Service Solutions (SPSS, version 2.0) (IBM SPSS Statistics for Windows, Armonk, NY), and the p-value was calculated. The p-value <0.05 was considered statistically significant.

## Results

A total of 65 (N) specimens from breast lesions were evaluated in the study, of which 26 (40%) were needle biopsies, 15 (23.07%) were lumpectomy specimens, and 24 (36.92%) were mastectomy specimens (Table 1).

**Table 1:** Types of specimen received (N=65)

Type of specimen	Numbers	Percentage (%)
Needle biopsies	26	40
Lumpectomy	15	23.07
Mastectomy	24	36.92

Among them, 60 cases presented with a lump in the breast, and five cases presented with nipple discharge. The majority of the cases were seen in the fifth decade. The mean patient age of presentation was 43.12.

Out of 65 cases, 24 cases were benign (37%), and 41 were malignant (63%). Among 24 benign lesions, 12 (50%) were fibroadenoma, five (20.83%) were intraductal papilloma, and seven (29.16%) were phyllodes tumor. Among 41 malignant cases, six (14.63%) were non-invasive carcinomas, and 35 (85.36%) were invasive carcinomas. Out of six non-invasive carcinomas, five (83.3%) were ductal carcinomas in situ, and one (16.6%) was encapsulated papillary carcinoma. The most common histological type of invasive carcinoma was invasive ductal carcinoma of no special type accounting for 72.22%. Other histological types were invasive ductal carcinoma with medullary features, invasive ductal carcinoma with metaplastic carcinoma, invasive lobular carcinoma, mixed invasive lobular and ductal carcinoma, invasive papillary carcinoma, and invasive papillary carcinoma with features of lobular carcinoma (Table 2).

**Table 2:** Histopathological types (N=65) n- Number %- Percentage

Histological			Diagnosis		n	%
Type	n	%				
Benign	24	36.92	Fibroadenoma		12	50
			Intraductal papilloma		5	20.83
			Phyllodes tumour		7	29.16
Malignant	41	63.07	Non-invasive (6)	DCIS	5	83.3
				Encapsulated papillary carcinoma	1	16.6
			Invasive (35)	Ductal carcinoma of no special type	2	72.22
				Invasive ductal carcinoma with medullary features	2	5.7
				Invasive ductal carcinoma with metaplastic carcinoma	1	2.8
				Invasive lobular carcinoma	4	11.4
				Mixed invasive lobular and ductal carcinoma	1	2.8
				Invasive papillary carcinoma	3	8.5
				Invasive papillary carcinoma with features of lobular carcinoma	1	2.8

Out of 41 malignant cases, seven (17.1%) showed Scarf-Bloom-Richardson grade I, 19 (46.34%) showed grade II, and 15 (36.58%) showed grade III (Table 3). Moreover, 11 cases (26.82%) showed metastatic lymph nodes.

**Table 3:** Histopathological grading of malignant cases (N=41)

Scarf Bloom Richardson grade		Grade I	Grade II	Grade III
Total (41)	In number	7	19	15
	In percentage	17.1	46.34	36.58

**Figure 1:**
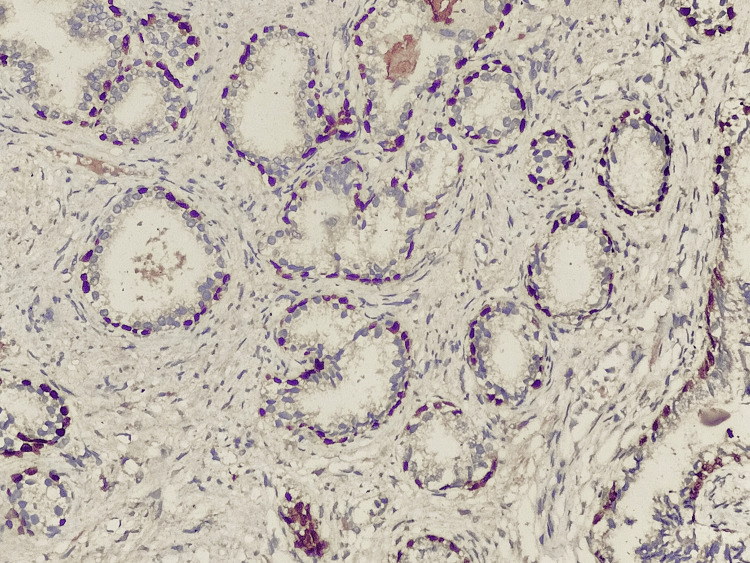
Photomicrograph of IHC in fibroadenoma showing nuclear positivity in myoepithelial cells (10x)

In all benign lesions, p63 immunoreactivity was seen in the myoepithelial cell layer surrounding the epithelial structures (Figure 1).

**Figure 2:**
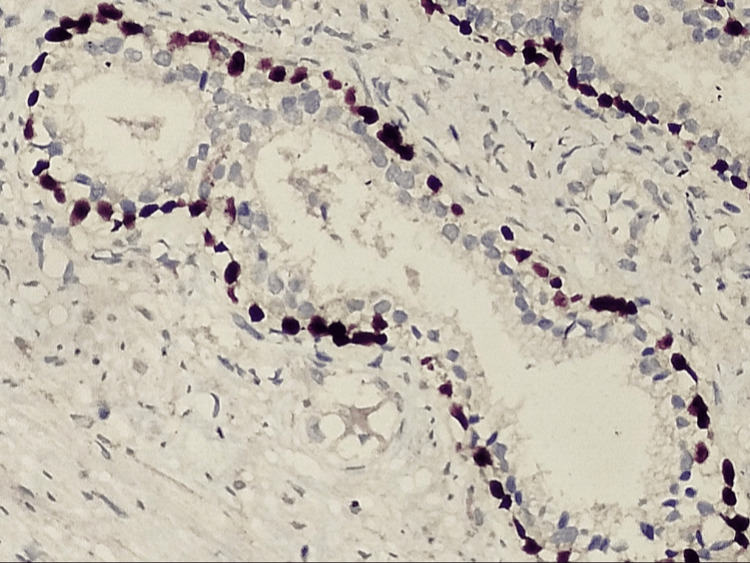
Photomicrograph of IHC in fibroadenoma showing nuclear positivity in myoepithelial cells (40x)

In most cases, the p63 expression was nuclear, and staining was in a continuous manner (Figure 2).

**Figure 3:**
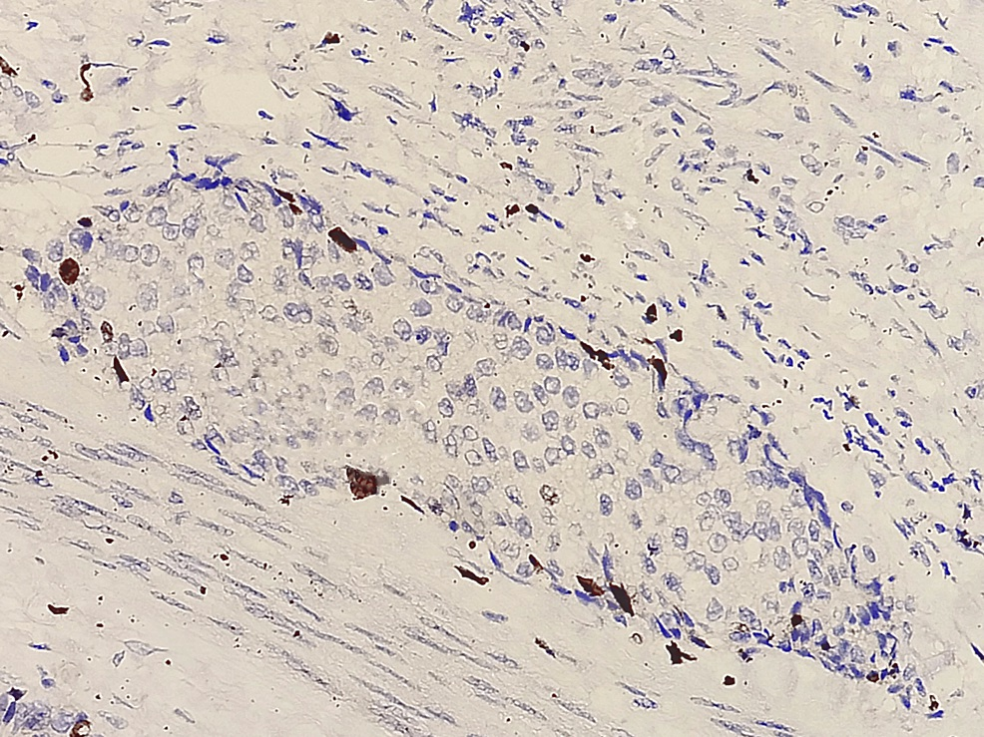
Photomicrograph of IHC in ductal carcinoma in situ showing discontinuous and less intensive staining in myoepithelial cells (40x)

p63 staining was also noted in a peripheral rim of myoepithelial cells in the in situ carcinomas. However, the staining intensity was less continuous than benign lesions (Figure 3).

**Figure 4:**
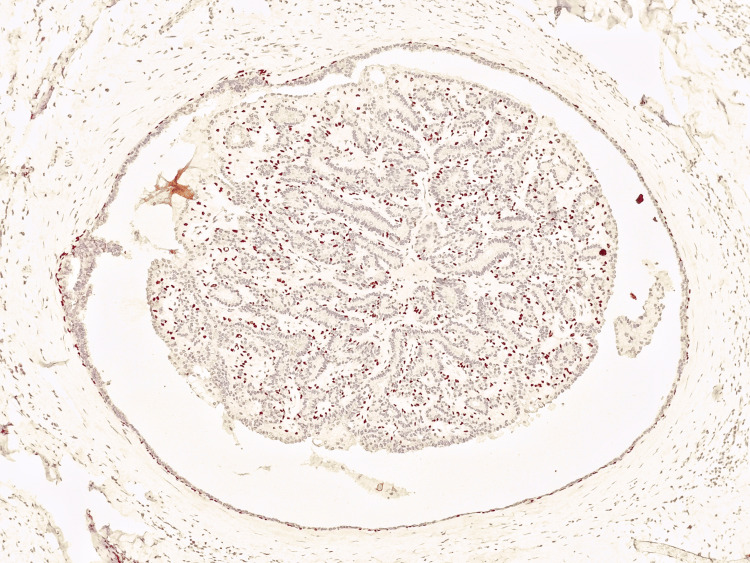
Photomicrograph of IHC in intraductal papilloma showing nuclear positivity in myoepithelial cells in fibrovascular cores of papillary fronds and periphery (10x)

The benign papillary lesions showed p63 staining in the fibrovascular core of the papillary fronds and at the periphery with mild attenuation at the dilated part (Figure 4).

**Figure 5:**
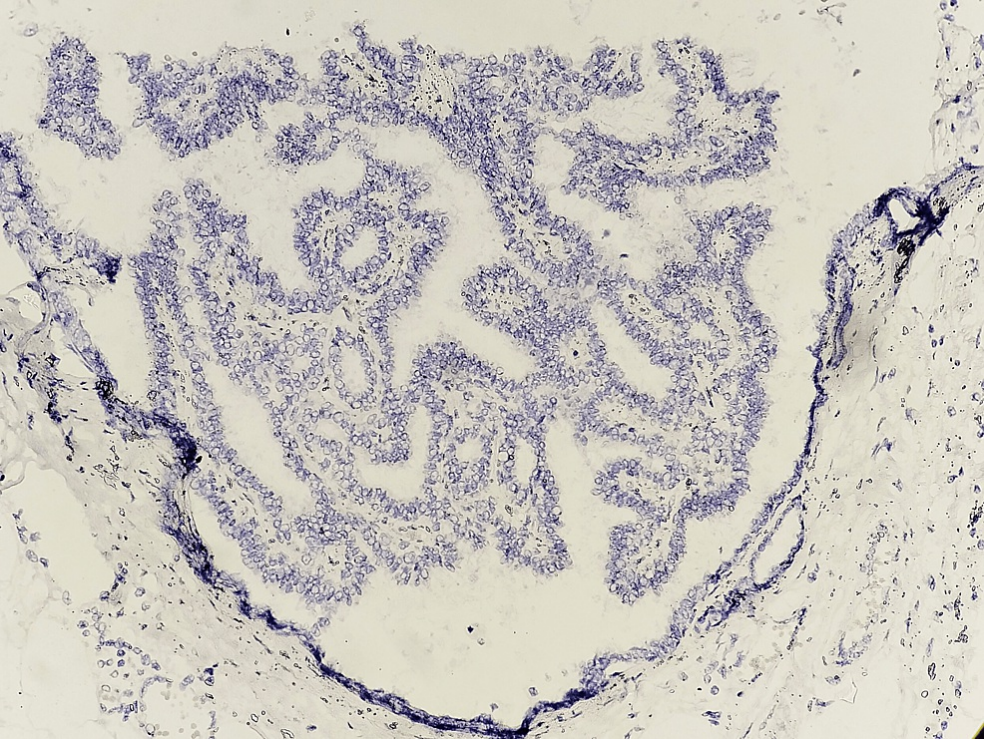
Photomicrograph of IHC in encapsulated papillary carcinoma showing a lack of p63 staining both in the fibrovascular core of the papillary fronds and in the periphery (20x)

Encapsulated papillary carcinoma showed a lack of p63 staining both in the fibrovascular core of the papillary fronds and in the periphery (Figure 5).

**Figure 6:**
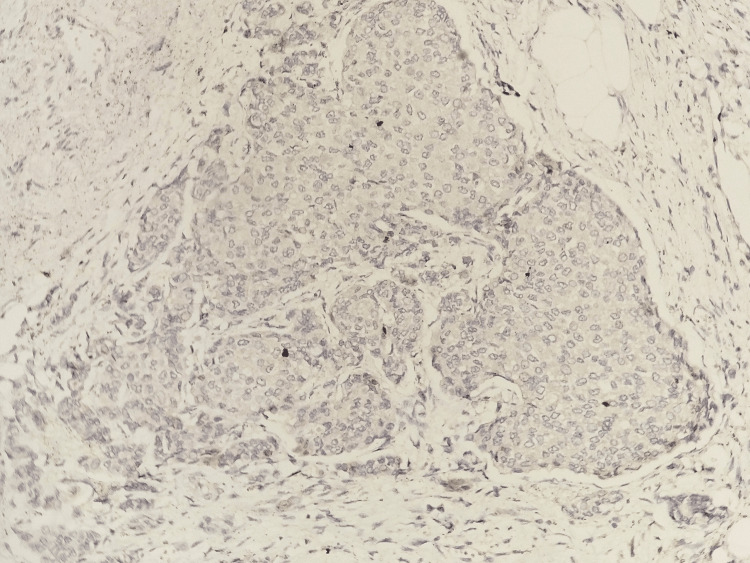
Photomicrograph of IHC in mixed infiltrating ductal and lobular carcinomas showing a negative staining pattern (20x)

All invasive breast carcinomas, irrespective of their histological type and grade, were devoid of p63 staining in the present study (Figure 6).

The difference in the staining pattern of p63 (Table 4) in benign lesions compared to non-invasive and invasive carcinomas was statistically significant (p-value is <0.001).

**Table 4:** p63 expression in benign and malignant cases (N=65)

Histological type	Continuous positivity and Strong intensity	Discontinuous positivity and weak intensity	Negative	p-value
Benign	24 (36.9%)			<0.001
Non-invasive		5 (83.3%)	1 (16.6%)	<0.001
Invasive			35 (85.3%)	<0.001

## Discussion

Breast carcinomas are treated based on clinical, radiological, and pathological findings. The presence or absence of invasion is a crucial histopathological feature that helps in the treatment aspect and prognosis. In rare cases, invasive foci may not be detected despite extensive microscopic examination and multiple histological sections of the entire breast tissue with routine staining. Moreover, metastases can develop without an invasion visible under routine microscopic examination. Various morphological criteria, including the presence of tubules, cytoplasmic vacuoles, dissociated tumor cells, the proliferation of fibroblasts, stromal infiltration by neoplastic cells, and the presence of myoepithelial cells around epithelial cell clusters, have been described [9-13]. Myoepithelial cells are elongated cells forming a continuous ring-like layer between the luminal epithelial layer and the basement membrane in the ductal cells; however, in lobules, they are stellular and discontinuous around the acini [10,12]. The role of myoepithelial cells in milk ejection during lactation is well-known. However, their role in proliferative neoplasms and invasive carcinomas must be explored further [11,14]. It is speculated that, as the myoepithelial cells secrete protease inhibitors and produce tumor-suppressive proteins, the loss of myoepithelial cells and their tumor-suppressive function leads to the transformation of ductal carcinoma in situ to invasive ductal carcinoma [10-16]. Several markers, such as basal cell-type cytokeratins, smooth muscle actin, calponin, and p63, can be used to demonstrate the presence of myoepithelial cells in breast lesions [14-16].

In the present study, all benign lesions showed immunohistochemical expression of p63 in the myoepithelial cell layer surrounding the epithelial cells. In all cases, p63 expression was nuclear, and staining was in a continuous manner. This was similar to studies by Ribeiro-Silva et al., where normal breast tissue and fibroadenomas showed p63 nuclear staining of a single continuous layer of cells surrounding the ductal and alveolar epithelium [2]. The benign papillary lesions showed p63 staining in the fibrovascular core of the papillary fronds and at the periphery with focal attenuation. Differentiation of papillary lesions of the breast is difficult, and the p63 marker can be very helpful. p63 has the highest sensitivity and lowest cross-reactivity and is easy to interpret nuclear positivity [17].

In our study, p63 also stained a rim of peripheral myoepithelial cells in DCIS, although the staining intensity was less continuous than benign lesions. No significant difference in staining was noted between high-grade and low-grade DCIS. This was similar to studies done by Khazai et al. [4] and Abdallah et al. [7]. The discontinuous staining in these cases may be due to ductal expansion associated with DCIS, resulting in fading of the myoepithelial cells. Another possibility is the presence of myofibroblasts within the stroma adjacent to nests of invasive carcinoma being misinterpreted as myoepithelial cells, resulting in a false-negative diagnosis [4]. The study done by Mohan et al. showed that a few high-grade DCIS cases did not show a myoepithelial layer around solid neoplastic nests with comedo necrosis. This suggests that the DCIS-like pattern represents invasive disease [10].

All invasive breast carcinomas, irrespective of their histological type and grade, were devoid of p63 staining in the present study, similar to other studies [16]. The assessment of invasion in malignant cases on routine hematoxylin and eosin staining was problematic due to peritumoral inflammation and fibrosis in two needle biopsies and one lumpectomy case in this study. However, negative p63 staining on immunohistochemistry and clinic-radiological correlation confirmed the diagnosis of invasive carcinoma in these cases.

Few studies showed that metaplastic carcinomas and rarely invasive ductal carcinomas stain positively for p63, but staining tends to be patchy and of less intensity than adjacent normal myoepithelial cells [2-3]. A study done by Ribeiro-Silva et al. showed that p63 was completely negative in neoplastic cells of low- and intermediate-grade invasive ductal carcinomas but was detected in high-grade invasive ductal carcinomas, suggesting the possibility that it is an indicator of the aggressiveness of breast carcinoma and that it may play a role in mammary tumorigenesis [2]. However, our study showed decreased p63 staining with the progression of ductal breast cancer. This suggests that p63 may be expressed in only a few aggressive tumors with certain histological types and further studies on its expression in high-grade invasive carcinomas may be required.

Of 41 malignant cases, two were papillary invasive carcinoma, which showed no p63 staining in the present study. However, studies by Stefanou et al. showed that p63 positivity could be seen in neoplastic cells of 33.3% of invasive papillary carcinomas, probably due to the exhibition of myoepithelial differentiation in these cells [11]. A study by Reisenbichler et al. concluded that breast papillary lesions have differential CK expression profiles that, especially in combination with p63, can be useful for their stratification, potentially also in needle biopsy material, in which more accurate and reproducible characterization is needed [18]. Myoepithelial cells may sometimes be difficult to identify in routine hematoxylin and eosin-stained sections, but they can be visualized with the use of immunohistochemical stains for myoepithelial antigens [19].

The findings of this study have to be seen in light of some limitations. The small sample size of the study may not be representative of the entire population, and the evaluation of p63 expression was not done on all histological subtypes of breast carcinomas.

## Conclusions

From this study, we can conclude that p63 is a useful marker for distinguishing benign epithelial lesions from carcinoma in situ on one end and invasive carcinoma on the other end. It can be judiciously used, in addition to hematoxylin and eosin-stained tissues, in identifying the myoepithelial cell layer in problematic cases and in papillary lesions of the breast. Its variable expression, intensity, and staining pattern seen in various studies suggest that it may have more significance at a molecular level in tumor genesis, in addition to staining the myoepithelial cells, and hence further studies are recommended.
